# Nutritional Risk Screening and Assessment

**DOI:** 10.3390/jcm8071065

**Published:** 2019-07-20

**Authors:** Emilie Reber, Filomena Gomes, Maria F. Vasiloglou, Philipp Schuetz, Zeno Stanga

**Affiliations:** 1 Department of Diabetes, Endocrinology, Nutritional Medicine and Metabolism, Bern University Hospital, and University of Bern, Freiburgstrasse 15, 3010 Bern, Switzerland; 2 The New York Academy of Sciences, 250 Greenwich Sweet, 40th floor, New York, NY 10007, USA; 3 Diabetes Technology Research Group, ARTORG Center for Biomedical Engineering Research, University of Bern, Murtenstrasse 50, 3008 Bern, Switzerland; 4 Medical University Department, Division of General Internal and Emergency Medicine, Kantonsspital Aarau, Tellstrasse 25, 5000 Aarau, Switzerland; 5 Department for Clinical Research, Medical Faculty, University of Basel, 4001 Basel, Switzerland

**Keywords:** nutritional risk screening, nutritional assessment, malnutrition

## Abstract

Malnutrition is an independent risk factor that negatively influences patients’ clinical outcomes, quality of life, body function, and autonomy. Early identification of patients at risk of malnutrition or who are malnourished is crucial in order to start a timely and adequate nutritional support. Nutritional risk screening, a simple and rapid first-line tool to detect patients at risk of malnutrition, should be performed systematically in patients at hospital admission. Patients with nutritional risk should subsequently undergo a more detailed nutritional assessment to identify and quantify specific nutritional problems. Such an assessment includes subjective and objective parameters such as medical history, current and past dietary intake (including energy and protein balance), physical examination and anthropometric measurements, functional and mental assessment, quality of life, medications, and laboratory values. Nutritional care plans should be developed in a multidisciplinary approach, and implemented to maintain and improve patients’ nutritional condition. Standardized nutritional management including systematic risk screening and assessment may also contribute to reduced healthcare costs. Adequate and timely implementation of nutritional support has been linked with favorable outcomes such as a decrease in length of hospital stay, reduced mortality, and reductions in the rate of severe complications, as well as improvements in quality of life and functional status. The aim of this review article is to provide a comprehensive overview of nutritional screening and assessment methods that can contribute to an effective and well-structured nutritional management (process cascade) of hospitalized patients.

## 1. Introduction

Nutrition is a basic need of life and thus plays an important role in health promotion and disease prevention. Nutritional intake and its controlling mechanisms (e.g., appetite, satiety) are highly complex physiological processes. These processes have a strong influence on nutritional status, which in turn depends on nutritional intake, its balanced supply of macro and micronutrients, and fluid intake. For various reasons, ill people may struggle to meet their nutritional and hydration requirements, and as a consequence, 20–50% of patients are malnourished or at high risk of malnutrition upon hospital admission [1]. One in five patients does not consume enough food to cover their energy or protein needs [2]. The underlying disease may directly impair nutritional intake and may induce metabolic and/or psychological disorders, which increase the nutritional needs or decrease food intake [3,4,5]. Frequent problems such as chewing and swallowing issues, immobility, and side effects of drugs and polypharmacy should not be underestimated in this regard [6,7]. A protracted decline in nutritional status results in a catabolic metabolism and chronic low-grade inflammation, potentially leading to several harmful consequences, such as loss of fat-free mass, immune dysfunction, higher complications and mortality rates, reduced quality of life, and prolonged hospital stays [8,9]. Malnutrition also influences the efficacy or tolerance of several treatments, such as antibiotic therapy, chemotherapy, radiotherapy, and surgery. The increased metabolism due to the stress of eventual surgical procedures further aggravates the nutritional metabolic risk, and is characterized by activation of the sympathetic nervous system, endocrine responses, and immunological and hematological changes—all leading to a hypermetabolic state, which may further increase patients’ nutritional needs. In addition, the fasting periods before many examinations and interventions, as well as inappropriate meal services, inadequate quality and flexibility of hospital catering, and insufficient assistance provided by the health care staff to the most vulnerable patients, lead to further inadequate food intake and deterioration of patients’ nutritional status.

Malnutrition should be considered and treated as an additional disease, as it has been shown to worsen clinical outcomes and to increase morbidity, mortality, and complication rates, thus causing additional costs [3,4,7,10,11,12,13,14]. However, malnutrition is preventable and mostly reversible with early adequate nutritional therapy. It often remains undetected due to lack of awareness, knowledge, and clinical protocols to identify and treat this problem within hospitals. The identification of malnutrition has typically been based on anthropometric, biochemical, and physical parameters, among others. However, there is currently no universally accepted gold standard (best method) for the assessment of nutritional status [15,16].

A systematic and standardized approach to identifying this condition is needed, and that is where nutritional screening tools play an important role [17]. When malnutrition is diagnosed, an individual nutritional care plan should be established by a nutrition specialist (e.g., dietitian, expert clinician) in consultation with a multidisciplinary team, and monitored regularly throughout the hospital stay. To improve the overall outcomes from nutritional treatment it is necessary to select patients with overt malnutrition, and those at most risk of developing nutritional deficiencies during their hospitalization. A systematic approach to addressing malnutrition in hospitals should start with the screening of all patients on admission, proceeding to a detailed assessment of nutritional status in those found to be at increased risk. In patients who are identified as malnourished or at nutritional risk, an appropriate nutritional intervention tailored to the individual patient’s needs should follow. Unfortunately, although the need for this process is well-recognized and forms part of several national and international guidelines, it is not carried out everywhere. In the well-known cross-sectional “NutriDay” survey conducted in 2007–2008, 21,007 patients from 325 hospitals in 25 European countries were included. Results showed that a screening routine existed in only half (53%) of the hospitals in the different regions, mostly performed with locally developed methods. While the routine screening of patients for malnutrition on hospital admission existed for 93% of units in the United Kingdom, less than 33% of units had this practice in Austria, Germany, and the South Eastern region. In addition, more than a quarter of all patients (27%) were considered to be at risk of malnutrition, and energy goals were not met in almost half (43%) of the surveyed population [18]. It remains necessary to raise awareness of malnutrition and to improve the outcomes of patients’ nutritional treatments.

We aimed to provide an extensive and critical overview of the nutritional screening and nutritional assessment methods of hospitalized patients, complemented by the description of the most novel technological approaches developed to improve the accuracy of dietary assessment. We hope that this review will be helpful to update clinicians involved in the nutritional care of this patient population.

## 2. Screening

Nutritional risk screening tools are very helpful in the daily routine to detect potential or manifest malnutrition in a timely manner. Such tools should be easy to use, quick, economical, standardized, and validated. Screening tools should be both sensitive and specific, and if possible, predictors of the success of the nutritional therapy. Nutritional screening should be part of a defined clinical protocol that results in a plan of action if the screening result is positive.

Diverse scores and screening systems were established in past decades for use in various clinical settings and patient populations (inpatients, community, geriatrics, etc.). Screening should be performed within the first 24–48 h after hospital admission and at regular intervals thereafter (e.g., weekly), in order to rapidly and accurately identify individuals who should be referred to the nutrition specialist (e.g., dietitian, expert clinician) for further assessment. Nutritional screening should include dynamic parameters rather than static ones—for example, recent weight loss, current body mass index (BMI), recent food intake, and disease severity. According to the systematic review conducted by van Bokhorst-de van der Schueren et al., at least 33 different nutritional risk screening tools exist [19]. The present work will use three as examples. The present work will use three examples thereof, which the European Society for Clinical Nutrition and Metabolism (ESPEN) recommends: the Nutritional Risk Screening 2002 (NRS-2002) for the inpatient setting, the Malnutrition Universal Screening Tool (MUST) for the ambulatory setting and the Mini Nutritional Assessment (MNA) for institutionalized geriatric patients [20].

One of the nutritional risk screening tools used most often in hospitals worldwide is the NRS-2002 (Table 1). The NRS-2002 was developed by Kondrup et al., and is meant to be a generic tool in the hospital setting—that is, useful in detecting most of the patients who would benefit from nutritional therapy [21]. This was recently shown in a large multicenter randomized controlled study in a medical inpatient population, which demonstrated a reduction of important clinical outcomes, including mortality, in patients at risk of malnutrition as determined by the NRS-2002 [22]. The NRS-2002 is a simple and well-validated tool which incorporates pre-screening with four questions. If one of these is answered positively, a screening follows which includes surrogate measures of nutritional status, with static and dynamic parameters and data on the severity of the disease (stress metabolism). For each parameter, a score from 0 to 3 can result. Age over 70 years is considered as a risk factor, and is included in the screening tool as well, giving 1 point. A total score of ≥3 points means that the patient is at risk of malnutrition or already malnourished and therefore a nutritional therapy is indicated. The NRS-2002 has been assessed and validated in hundreds of studies, including randomized controlled trials, and has been shown to be very reliable if administered by trained staff.

The MUST (Table 2) was developed to identify malnourished individuals in all care settings (hospitals, nursing homes, home care, etc.) [23]. It was the basis for the NRS-2002 [21]. Recent food intake is not included, and calculations of the weight loss percentage may be a barrier for the busy healthcare staff on the wards.

The MNA is the screening tool most frequently used in institutionalized geriatric patients (Table 3). It combines screening and assessment features. Unlike the NRS-2002, the MNA includes diverse components (loss of appetite, altered sense of taste and smell, loss of thirst, frailty, depression) often relevant for the nutritional status of older people. It also includes anthropometric measurements, nutritional habits, general condition, and self-evaluation. Both the MNA (complete form) as well as a short-form MNA (MNA-SF) are available. The complete MNA includes eighteen items in four domains (Appendix A). The MNA-SF includes only six items, but is quicker and as effective as the long version. If the total score is 11 points or less, the patient is considered at risk of malnutrition or malnourished and the full version (assessment) should be performed.

It is important for clinicians to understand how the tools were validated and for which population and care setting they were developed in order to determine if the tool is appropriate for use in their institution [24]. For example, a study that aimed to identify the most appropriate nutritional screening tool for predicting unfavorable clinical outcomes in 705 patients admitted to a Brazilian hospital compared the performance of NRS-2002, MNA-SF, and MUST. The authors observed that the NRS-2002 and MNA-SF had similar performance in predicting complications, very long length of hospital stay, and mortality, but the NRS-2002 had the best yield, and therefore recommended the use of this tool in the Brazilian inpatient population [25].

## 3. Assessment

Nutritional assessment should be performed in patients identified as at nutritional risk according to the first step (i.e., screening for risk of malnutrition). Assessment allows the clinician to gather more information and conduct a nutrition-focused physical examination in order to determine if there is truly a nutrition problem, to name the problem, and to determine the severity of the problem [26]. The data collected in a nutritional assessment are often similar to data collected in the screening process, but in more depth. Screening assesses risk whereas assessment actually determines nutritional status [26]. The observation and documentation of oral nutritional intake, including qualitative and quantitative aspects, and measurement of energy, protein, and micronutrient intake, is an important part of nutritional assessment.

There is a limited number of tools used for the assessment of nutritional status. The most-used tool is the Subjective Global Assessment (SGA), which includes information on a medical history (weight loss; dietary intake change; gastrointestinal and functional impairment) and physical examination (loss of subcutaneous fat; muscle wasting; ankle edema, sacral edema, and ascites). Each patient is classified as either well nourished (SGA A), moderately or suspected of being malnourished (SGA B), or severely malnourished (SGA C). A limitation of using SGA is that it only classifies subjects into three general groups, and it does not reflect subtle changes in nutritional status. Furthermore, it is subjective, does not account for biochemical values (e.g., visceral protein levels), and its sensitivity, precision, and reproducibility over time have not been extensively studied in some patient populations. Thus, here we describe the several components that should be part of the nutritional assessment process and interpreted by specialized clinical staff (e.g., dietitians) [27,28,29].

Most of these components have limited sensitivity and specificity when used individually; therefore, methods for identifying malnourished patients require the use of several parameters and the clinical judgment of experienced and specialized clinical staff. Detailed evaluation leads to an understanding of the nature and cause of the nutrition-related problem, and will inform the design of a personalized nutritional care plan [30].

### 3.1. Anthropometric Measurements

#### 3.1.1. Body Weight and Body Mass Index

Body weight, height, and the resulting BMI are important parameters which are relatively easy to obtain from patients with acute as well as chronic diseases. If height cannot be assessed (e.g., in bedridden patients or patients that are unable to stand), knee height or demi-span (also recommended by the MNA) may be used to estimate height by means of standard formulas [31,32]. The body weight measurement should be standardized (e.g., measured at the same time of day and with the same amount of light clothing) to obtain a reliable weight trend. The BMI is an indicator of chronic malnutrition. Europeans are considered underweight when BMI is <18.5 kg/m^2^. BMI values under this cutoff are associated with poor outcome and higher mortality rates, as are BMI values greater than 30 kg/m^2^ (typically classified as obesity). In older adults the cut-off for the definition of underweight is higher, that is, <22 kg/m^2^, as carrying some extra weight seems to be protective in this population. However, the BMI has some limitations. For example, it may be biased by fluid overload and edemas, and does not describe body composition (for example, a high BMI can be seen in fat individuals and also in very muscular athletes). Thus, the BMI does not reflect potentially pathological weight loss nor the patient’s actual food intake. Unintentional weight loss is paramount for the assessment of nutritional status, as it points to a catabolic metabolic situation and is associated with higher morbidity and mortality rates.

#### 3.1.2. Skinfold Measurements

One of the easiest and lowest-priced non-invasive methods is the measurement of the circumference of a limb (e.g., mid-arm, calf) and of skinfold thickness (SFT). The subcutaneous fat tissues normally account for half of the entire body fat mass, and the measurement of SFT gives information on the energy stores of the body, mainly fat stores (i.e., triglycerides). To estimate the total amount of body fat, four skinfolds need to be measured [33]:

– Biceps skinfold (front side of the middle upper arm);
– Triceps skinfold (back side of the middle upper arm);
– Subscapular skinfold (under the lowest point of the shoulder blade); and
– Suprailiac skinfold (above the upper bone of the hip).

The measurement of SFT requires trained staff and defined conditions. The high interindividual variability is a clear disadvantage of this method, as age, gender, and ethnicity influence the fat mass. The mid-upper-arm muscle circumference (MAMC) reflects the muscle mass, while the mid-arm muscle area (MAMA) gives information about the muscle protein stores, as half of the body’s proteins are stored in the skeletal muscles. The MAMA is calculated from the MAMC and the triceps SFT (MAMA = MAMC − (0.314 × SFT)). The decrease in MAMA shows the loss of muscle mass, as a mobilization of the endogenous proteins. This method is not reliable in patients with fluid overload, however, nor does it represent short-term modifications of the nutritional status. The reliability of both the SFT and the MAMA strongly depend on the reference values. For these reasons, triceps skin fold and MAMA are mostly used for research purposes and not in daily clinical routine, as they give validated data—especially when measurements are performed by the same investigator and repeated in a given time period.

#### 3.1.3. Body Composition

**Figure 1:**
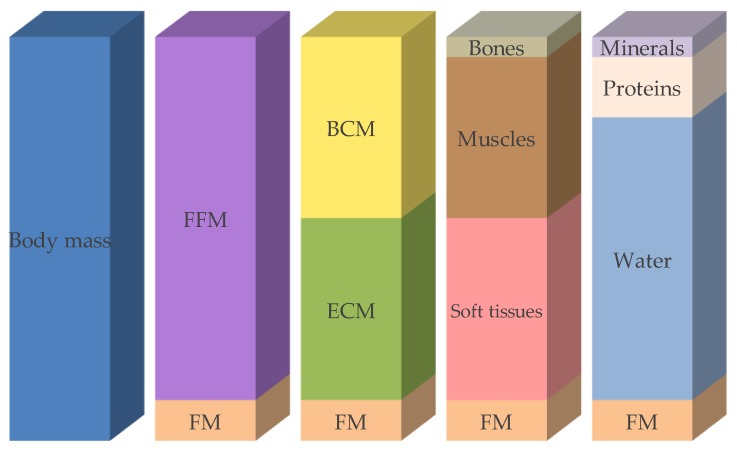
Compartment models of body composition. FFM: fat-free mass, FM: fat mass, BCM: body cell mass, ECM: extracellular cell mass. Modified after [40].

Body weight—including weight loss, calculation of the BMI, and measurement of the length, circumference, or thickness of various body parts—is useful for the assessment of nutritional status. Body composition describes the body compartments, such as fat mass, fat-free mass, muscle mass, and bone mineral mass, depending on the body composition model used (Figure 1). Body composition measurements may serve as an early diagnostic tool, as quantification, or as a follow-up method that helps to assess nutritional status [34]. Such measurements contribute to the diagnosis of sarcopenia and sarcopenic adiposity, and may establish reference values (energy expenditure/kg fat-free mass (FFM) or power/g muscle). Body composition may change due to disease, age, physical activity, and starvation. There are several methods available to determinate body composition, more or less invasively, as described in the following section (Table 4).

#### 3.1.4. Bioelectrical Impedance Analysis (BIA)

Bioelectrical impedance analysis (BIA) is a simple, inexpensive, non-invasive method of estimating body composition. It is suitable for bedside measurements which depend on the body’s proportions of fat, muscle, and water. BIA relies on the conduction of an alternating electrical current by the human body. The current passes easily through tissues containing a lot of water and electrolytes like blood and muscles, whereas fat tissues, air, and bone are harder to pass through. Therefore, the larger the fat-free mass, the greater the capacity of the body to conduct the current. BIA gives good information about total body water, body cell mass, and fat mass when correcting for age, sex, and ethnicity. However, BIA is not recommended in patients with fluid overload, in patients at extremes of BMI (<16 or >34 kg/m^2^), in intensive care unit patients, or in the elderly [35,36]. The newly developed bioelectrical impedance vector analysis (BIVA) provides information about hydration status, body cell mass, and cell integrity through the vector length and position. Both malnutrition and obesity are clearly reflected by BIVA, making it attractive to assess and monitor patients’ nutritional status.

#### 3.1.5. Creatinine Height Index (CHI)

Creatine is metabolized to creatinine at a more or less stable rate, and reflects the amount of muscle mass [37]. Creatinine excretion correlates with lean body mass and body weight. The creatinine height index (CHI) [38] is a measure of lean body mass and is calculated as follows: CHI (%) = measured 24 h urinary creatinine × 100/normal 24 h urinary creatinine. Urinary creatinine excretion may be influenced by several factors, such as renal insufficiency, meat consumption, physical activity, fever, infections, and trauma. Additionally, the collection of 24-h urine is challenging in daily practice and further limits the use of this method.

#### 3.1.6. Dual Energy *X*-ray Absorptiometry (DXA)

DXA is currently considered the gold standard of body composition measurement. It is increasingly used in clinical practice and in research, despite some exposure to radiation. DXA depends on radiological density analysis (usually in the hip and spine) and is a useful, indirect method of measuring fat mass, fat-free mass, and bone mineral mass.

#### 3.1.7. Magnetic Resonance Tomography (MRT) and Computed Tomography (CT)

Magnetic resonance tomography (MRT) and computed tomography (CT) allow the quantification of fat mass and fat-free mass, giving information about the fat distribution and enabling an estimation of skeletal muscle mass. Unlike CT, MRT does not require ionizing radiation. These two methods are mainly used in research due to their restricted availability, their cost, and the time expended [39]. However, it is often possible to obtain nutritional information from scans taken for general diagnostic purposes.

#### 3.1.8. Further Methods Used to Measure Body Composition

Several other methods are available, mainly for research purposes due to their complexity. These demanding and expensive methods include air displacement plethysmography (ADP), dilution methods, the measurement of total body potassium, and in vivo neutron activation analysis [40].

Air displacement plethysmography (ADP) is a method to determine the body density (body weight/body volume). It is based on the determination of the body volume by means of air displacement having regard to the residual air volume in the lungs and the gastrointestinal tract. Since the density of fat differs from the density of fat free mass, they can both be determined using a two-compartment model. ADP may also be used in ill patients, unlike other densitometry measurement using hydrodensitometry.

The dilution methods aim to determinate the total body water by means of dilution of non-radioactive isotopes (e.g., deuterium). Such tracers are given orally or parenterally, and their concentrations in urine and blood are measured after a defined time. Extracellular water can then be determined using bromide or sulfate, allowing the definition of intracellular water.

Since potassium is mostly found intracellularly and the natural isotope K^40^ is present in constant fraction, the measurement of the potassium allows the calculation of the body cell mass and thus enables the very accurate determination of the body cell mass.

With the in vivo neutron activation, the body is irradiated with neutron radiation, inducing the emission of a characteristic spectrum of gamma-radiations. This expensive method allows the quantification of single elements such as nitrogen, calcium, sodium, etc.

### 3.2. Biochemical Analysis

There is no single parameter that can thoroughly assess nutritional status or monitor nutritional therapy. However, a set of laboratory parameters in the clinical routine (e.g., complete blood count, lipid profile, electrolytes, liver parameters) may provide valuable information about a patient’s nutritional status (e.g., proof of nutrient deficiency, information about the etiology of malnutrition, follow-up nutritional therapy), about the severity and activity of the disease, and about changes in body composition (Table 5) [41]. Laboratory values—particularly in chronically malnourished patients—may help to detect deficiencies in vitamins (C, D, E, K, thiamine, B6, B12, and folic acid) and trace elements (zinc, selenium, and iron) and help to monitor current substitution therapies. In the early phase of refeeding, potassium, phosphate, and magnesium deficiencies may occur, potentially leading to severe complications (e.g., refeeding syndrome); hence, there is a need for close monitoring of these electrolytes.

Laboratory values are mostly delayed and costly, and largely dependent on the analytic method and the analyzing laboratory. Additionally, numerous non-nutrition-related factors may influence the laboratory parameters (e.g., inflammatory markers such as CRP), leading to distorted values. Thus, laboratory values must always be interpreted within the clinical context.

### 3.3. Clinical Evaluation

#### 3.3.1. Patient Clinical History

The patient’s clinical history is a subjective and retrospective description of the patient’s condition. It is the starting point of the nutritional assessment. Factors leading to malnutrition such as pain, gastrointestinal symptoms (e.g., diarrhea, vomiting, constipation), weight loss, loss of appetite, inability to chew or swallow, and poor dentition/oral health are discussed with the patient. The patient’s clinical history should include previous medical condition (chronic or acute disease, symptoms of psychiatric illness, presence of conditions that may lead to metabolic stress (e.g., infection), as well as the actual functional capacity and physiological changes possibly influencing nutritional requirements or body composition (e.g., loss of muscle mass).

#### 3.3.2. Physical Examination

Physical examination is an objective method of detecting clinical signs and symptoms of nutritional deficiencies of vitamins and minerals (e.g., poor muscle control, night vision impairment, vertical lip cracks, depression), and allows the assessment of tolerance to nutritional support (e.g., abdominal distention, vomiting, diarrhea) [42]. Some clinical signs are specific to a specific disease or nutrient deficiency. Others are non-specific and need further tests to elucidate their etiology (Table 6). Physical examination includes the control of vital parameters, the inspection and palpation for water retention (edema and ascites), and a rough assessment of muscle mass and subcutaneous fat stores.

#### 3.3.3. Physical Function

Functional measurements are increasingly important in nutritional assessment. Indeed, muscle strength and cognitive functions all influence quality of life. Energy deficiency diminishes muscle strength and power, as well as overall physical condition. It is therefore very relevant to have information about muscle function and strength in the clinical setting. Muscle function tests are very sensitive to nutritional deficiencies, and therefore also to nutritional interventions. Changes can therefore be noticed much earlier than through body composition tests, for example. Hand dynamometry has been validated as a nutritional marker, correlates very well with the nutritional status, and is simultaneously a good predictor of surgical outcome, increased hospital length of stay, higher re-hospitalization rates, and decreased physical status. It is additionally a good predictor for short- and long-term mortality [43]. This test is easy, quick, and low-priced, but largely depends on the patient’s cooperation. Other possible measurements are knee extension, hip flexion strength, or peak expiratory flow. Measurement of the distance walked in a given time (e.g., at a 4-m gait speed) may also provide good information on the global condition [44].

#### 3.3.4. Medication

A patient’s prescribed medications (including vitamin/mineral/botanical supplements) should be examined regarding potential drug–nutrient interactions and nutrition-related side effects (interactions with appetite, gastrointestinal function or symptoms).

### 3.4. Dietary History, Current Dietary Intake, and Innovative Dietary Assessment Methods

The dietary history includes the patient’s dietary habits and preferences, including cultural and religious habits, special diets, as well as food allergies or intolerances. Fluid and alcohol intake should also be recorded.

The energy and protein balance and the comparison between food intake and energy expenditure reflect the current nutritional status—whether the patient’s dietary intake is sufficient or not.

The quantification of food intake is one of the key approaches to assessing nutritional risk in individual patients. The assessment of macronutrients (fat, carbohydrates, and proteins) is as important as the assessment of micronutrients (vitamins, trace elements). There are numerous standardized methods of measuring food intake, such as 24 h food recall, food frequency questionnaires, and direct observation (food records are frequently used by nurses for institutionalized patients). These provide (semi-) quantitative information. The accurate assessment of food intake is difficult and error-prone. There is a growing need for more accurate dietary assessment methods. High-quality data are essential for research on the association between diet and health, for an understanding of dietary patterns, and for the identification of nutrition-related health problems [45].

Innovative technologies that improve dietary assessment have been proposed recently, and can be classified into four principal groups according to the technological features that each of them incorporate [46,47,48,49,50]:

– *Manual dietary assessment*—The user inserts all required data (e.g., portion size estimation, type of food) on a web page, smartphone app, etc. [50]. This method replaces the paper-based methods of dietary assessment into an electronic form by the use of pictures, video, text, or voice without the inclusion of automatic features.
– *Dietitian-supported assessment—*The user takes photos of the food and sends them to the dietitian. These data are then analyzed by nutrition experts who use standardized methods (e.g., nutritional software) to estimate the corresponding amount of nutrients [51]. No automation features are usually incorporated.
– *Wearable devices monitoring food intake—*Devices that directly measure eating behavior [52], such as detection systems which identify eating gestures (ear-based chewing and swallowing) in order to complement self-reporting of nutrient intake.
– *Computer-aided assessment—this includes*:
  (i) Systems that incorporate some degree of automation. These either use bar-code readers in order to automatically recognize packaged food labels [50], or utilize smartphone applications that integrate the automatic recognition of food items. In this case, the user takes photos of the food and the system recognizes the type of food. Typically, in this situation the user needs to manually insert or select the volume/portion of the food items in order for the system to be able to translate the information into macronutrients and energy [53].
  (ii) Systems that are completely based on artificial intelligence. In a typical scenario, the user takes photo(s) of the food and then the system automatically and in real-time identifies the different food items (identification), recognizes the type of each of them (labeling), and creates a 3D model of each of them (3D reconstruction) [54,55,56,57,58]. Supported by food composition databases, food images are translated into nutrient values such as grams of macronutrients or calories [54,56].

These new technologies have several advantages. They do not (fully) rely on a respondent’s memory; they are based on a number of automatic data-processing steps, thus minimizing user-related variability [45]; there is minimal participant burden; and there are reduced research and administrative costs [50]. Additionally, these technologies offer portability and greater social acceptability than paper-based methods [59]. Some additional advantages of computer-aided methods include decreased workload and costs (excluding costs for software development) [48], minimization of researchers’ transcription errors [60], reduced paper waste and postage costs, and the optimization of space, security, and organization required for paper file storage [61].

However, there are also some limitations for each group. The manual dietary assessment methods provide all the disadvantages of paper-based methods except for expenditures related to paper usage. Body sensor monitoring provides no input about the type or quality of the food that is captured [50]. What is more, dietician-supported assessment is labor-intense and expensive to analyze [50]. Moreover, with the AI-based systems, it is not possible to capture all the basic nutrient information (including cooking methods) with one single image [45], and the majority of the existing apps are manual or semi-automatic in terms of food logging, and non-automatic in portion size estimation. Individuals tend to estimate portion size inaccurately [62]; almost half of the errors found in food records are attributed to such faulty estimations [63]. Other possible disadvantages are under-reporting due to either poor image quality or user negligence in taking an adequate number of pictures before and after food and drink consumption [64]. In addition, some food types such as mixed foods or liquids are difficult to analyze with automated image analysis [58]. Tools that include only some AI components are usually non-validated; they include a limited number of food categories, and questions relating to the used nutrient databases arise [50]. The most important limitation of the majority of these technologies is the need for a tech-savvy user [45].

Several studies have compared dietary assessment by traditional methods versus innovative technologies. Some of them conclude that electronic records would be a useful tool, both for large-scale epidemiological studies and in the clinical context [61]. Others conclude that apps could replace the traditional 24-h recall and serve as feasible tools for dieticians investigating dietary intake at a population level [65]. The longer the app recording periods are, the better the correlation between the traditional and the innovative methods seems to be [66]. However, novel technologies for dietary assessment appear valid at the population level rather than for individualized support [67,68,69]. Even though there are an increasing number of studies in the domain of innovative technologies, sample sizes are relatively low, and duration is usually short. Therefore, there is a need for well-designed long-term studies to explore and analyze the combination of traditional methods and state-of-the-art technological tools which characterizes the new era of nutritional assessment.

Energy requirements are calculated from the basal energy requirement multiplied by an activity factor. They can be calculated with formulae (e.g., the Harris–Benedict formula [70]) or through a simplified general rule based on energy values between 25–35 kcal per kg of body weight per day, with adjustment for underweight and overweight patients (30 × body weight, +20% if BMI <20 kg/m^2^ or −20% if BMI >30 kg/m^2^) [71]. These formulae cannot be used in special situations (e.g., in ICU patients). The protein requirement may be estimated by using 1.2–1.5 g/kg body weight per day (0.8 g/kg/d in case of chronic kidney failure) [22]. The specific macronutrient requirements are described in Table 7. Indirect calorimetry remains the gold standard for the assessment of energy requirements, but in many clinical settings this option is not available, as indirect calorimeters may not be easy to operate and may not be portable or affordable.

Several conditions may impair food intake and should be taken into account as well. Among these are chewing and/or swallowing problems and functional limitations impairing independent eating. Additionally, cognitive changes affecting appetite and ability to feed oneself, and physiological changes that affect the desire to eat, may negatively impact the dietary intake.

### 3.5. Quality of Life

The assessment of quality of life is a more subjective parameter that is being increasingly included in nutritional assessment. It reflects the current health status, and may be used as an outcome parameter to monitor nutritional therapy. It is based on the perception of wellbeing in different domains—for example, symptoms (pain), physical (mobility, strength), psychological (anxiety, depression), and social (isolation), all potentially having an effect on eating. There are many questionnaires available, but there is no established consensus on which should optimally be used.

## 4. Conclusions and Outlook

Malnutrition is a frequent threat in hospitals, and is associated with negative outcomes. However, it remains a mostly treatable condition when there is adequate nutritional management. It is crucial to identify patients who are at nutritional risk or malnourished as early as possible, allowing the start of timely and effective nutritional support. Identifying patients at risk of malnutrition is the first step in the nutritional care process within a multimodal care system. Nutritional risk screening with simple and rapid tools should be performed systematically in each patient at hospital admission to detect patients who are nutritionally at risk or malnourished. Comprehensive detailed nutritional assessment should be performed thereafter in those patients identified as at risk of malnutrition or who are malnourished. This screening should be performed by a specialist (e.g., a dietician) using subjective and objective parameters such as clinical history, physical examination, body composition measurements, functional assessment, and laboratory values. New assessment methods may be very helpful, as they are accurate and quick. A nutritional care plan should be drawn up using an interdisciplinary approach and implemented to improve the patient’s condition. Systematic nutritional risk screening and standardized nutritional management may also contribute to reduced healthcare costs.

## Figures and Tables

**Table 1:** Nutritional Risk Screening 2002. APACHE: acute physiology and chronic health evaluation; BMI: body mass index; COPD: chronic obstructive pulmonary disease; ONS: oral nutritional supplement.

**Pre-Screening**			
Is the BMI of the patient < 20.5 kg/m^2^			Yes
Did the patient lose weight in the past 3 months?			Yes
Was the patient’s food intake reduced in the past week?			Yes
Is the patient critically ill?			Yes
If yes to one of those questions, proceed to screening.			
If no for all answers, the patient should be re-screened weekly.			
**Screening**			
Nutritional status	score	Stress metabolism (severity of the disease)	score
None	0	None	0
Mild Weight loss >5% in 3 months OR 50–75% of the normal food intake in the last week	1	Mild stress metabolism	1
		Patient is mobile Increased protein requirement can be covered with oral nutrition *Hip fracture, chronic disease especially with complications* e.g., *liver cirrhosis, COPD, diabetes, cancer, chronic hemodialysis*	
Moderate	2	Moderate stress metabolism	2
Weight loss >5% in 2 months OR BMI 18.5–20.5 kg/m^2^ AND reduced general condition OR 25–50% of the normal food intake in the last week		Patient is bedridden due to illness Highly increased protein requirement, may be covered with ONS *Stroke, hematologic cancer, severe pneumonia, extended abdominal surgery*	


Severe Weight loss >5% in 1 month OR BMI <18.5 kg/m^2^ AND reduced general condition OR 0–25% of the normal food intake in the last week	3	Severe stress metabolism Patient is critically ill (intensive care unit) Very strongly increased protein requirement can only be achieved with (par)enteral nutrition *APACHE-II >10, bone marrow transplantation, head traumas*	3

Total (A)		Total (B)	
**Age**			
<70 years: 0 pt			
≥70 years: 1 pt			
**TOTAL = (A) + (B) + Age**			
≥3 points: patient is at nutritional risk. Nutritional care plan should be set up			
<3 points: repeat screening weekly			

**Table 2:** The Malnutrition Universal Screening Tool.

**Malnutrition Universal Screening Tool (MUST)**				
BMI (kg/m^2^)		Unintentional weight loss in the past 3–6 months		Acute illness with reduced food intake (estimated) for ≥5 days
≥20	0	≤5%	0	No = 0
18.5–20.0	1	5–10%	1	Yes = 2
≤18.5	2	≥10%	2	
**Overall Risk for Malnutrition**				
Total	Risk	Procedure	Implementation	
0	Low	Routine clinical care	Clinic: weekly	
			Nursing home: monthly	
			Outpatient: yearly in at-risk patient groups, e.g., age >75 years	
1	Medium	Observe	Clinic, nursing home, and outpatient:	
			Document dietary intake for 3 days.	
			If adequate: little concern and repeat screening (hospital weekly, care home at least monthly, community at least every 2–3 months).	
			If inadequate: clinical concern. Follow local policy, set goals, improve and increase overall nutritional intake, monitor and review care plan regularly.	
≥2	High	Treat	Clinic, nursing home, and outpatient:	
			Refer to dietitian, Nutritional Support Team, or implement local policy. Set goals, improve and increase overall nutritional intake. Monitor and review care plan (hospital weekly, care home monthly, community monthly).	

**Table 3:** The Mini Nutritional Assessment Short-Form.

**Screening**		
A	Has food intake declined over the past 3 months due to loss of appetite, digestive problems, or chewing or swallowing difficulties?	0 = severe decrease in food intake
		1 = moderate decrease in food intake
		2 = no decrease in food intake
B	Weight loss during the last 3 months	0 = weight loss greater than 3 kg
		1 = does not know
		2 = weight loss between 1 and 3 kg
		3 = no weight loss
C	Mobility	0 = bedridden or chair bound
		1 = able to get out of bed/chair but does not go out
		2 = goes out
D	Has the patient suffered psychological stress or acute disease in the past 3 months?	0 = yes
		2 = no
E	Neuropsychological problems	0 = severe dementia or depression
		1 = mild dementia
		2 = no psychological problems
F1	Body mass index (BMI)	0 = BMI less than 19
		1 = BMI 19 to less than 21
		2 = BMI 21 to less than 23
		3 = BMI 23 or greater
*If BMI is not available, replace question F1 with F2. Do not answer F2 if F1 is already completed.*		
F2	Calf circumference (CC) in cm	0 = CC less than 31
		3 = CC 31 or greater
**Screening Score**		
12–14 points	Normal nutritional status	
8–11 points	At risk of malnutrition	
0–7 points	Malnourished	

**Table 4:** Advantages and disadvantages of methods used to assess body composition.

Method	Target	Precision	Expenditure (Time/Apparatus)	Costs
Anthropometrics	FM, fat distribution, MM	↓	↓	↓↓
Bioelectrical impedance analysis	TBW, FM, FFM, BCM phase angle	↑	↓	↓
Creatinine height index	MM	↓	-	↓
Dual energy *X*-ray absorptiometry	FM, bone mineral content, soft tissues, bone density	↑	↑	↑
Magnetic resonance tomography	MM, FM, fat distribution	↑	↑	↑↑
Computed tomography	FM, fat distribution, MM	↑	↑	↑
Dilution method	TBW, FM, FFM (deuterium) ECW, ICW (bromide)	↑	↑	-
Potassium count	BCM, FFM, FM	↑	↑	↑↑
Neutron activation	Ca, Na, Cl, PO_4_, N, H, O, C	↑	↑	↑↑

FM: fat mass; FFM: fat-free mass; MM: muscle mass; TBW: total body water; BCM: body cell mass; ECW: extracellular water; ICW: intracellular water; Ca: calcium; Na: sodium; Cl: chloride; PO_4_: phosphate; N: nitrogen; H: hydrogen; O: oxygen; C: carbon.

**Table 5:** Laboratory values to detect malnutrition and monitor nutritional status [41].

Laboratory Value	Nutrition Independent Factors	Half-Life	Appropriateness to Detect Malnutrition	Appropriateness to Monitor Nutritional Therapy
Albumin	↑ dehydration	20 d	+/++	Not appropriate due to high suggestibility and long half-life
	↓ inflammation, infections, trauma, heart failure, edema, liver dysfunction, nephrotic syndrome			
			Not appropriate in case of anorexia and acute illness	
Transferrin	↑ renal failure, iron status, acute hepatitis, hypoxia	10 d	+	+
	↓ inflammation, chronic infections hemochromatosis, nephrotic syndrome, liver dysfunction		Low sensitivity and specificity	Concentration is independent of the energy and protein intake
Prealbumin/Transthyretin (TTR)	↑ renal dysfunction, dehydration, corticosteroid therapy	2 d	++	++/+++
			Not appropriate to detect anorexia Subnormal values within one week in case of fasting	
				One of the most appropriate proteins
	↓ inflammation, hyperthyreosis, liver disease, overhydration			
Retinol binding protein (RBP)	↑ kidney failure, alcohol abuse	12 h	Idem prealbumin	Idem prealbumin
	↓ hyperthyreosis, chronic liver diseases, vitamin A deficiency, selenium deficiency			
Insulin-like growth factor 1 (IGF-1)	↑ kidney failure	24 h	++	+++
				More specific than retinol-binding protein and prealbumin/transthyretin
	↓ liver diseases, severe catabolic status, age		Rapid decrease in fasting periods	
Urinary creatinine	↑ collection time >24h, infection, trauma	-	1 mmol of creatinine is derived from 1.9 kg of skeletal muscle mass	Not appropriate, very slow
	↓ insufficient collection time, acute kidney failure			
Lymphocytes	↑ healing phase after infection, hematologic diseases	-	+	Not appropriate, very slow
	↓ sepsis, hematologic disease, immune suppressants, steroids		Very unspecific	

**Table 6:** Clinical signs and symptoms of micronutrient deficiencies [40,42].

Body Region	Signs	Possible Deficiencies
Skin	Petechiae	Vitamins A, C
	Purpura	Vitamins C, K
	Pigmentation	Niacin
	Edema	Protein, vitamin B1
	Pallor	Folic acid, iron, biotin, vitamins B12, B6
	Decubitus	Protein, energy
	Seborrheic dermatitis	Vitamin B6, biotin, zinc, essential fatty acids
	Unhealed wounds	Vitamin C, protein, zinc
Nails	Pallor or white coloring Clubbing, spoon-shape, or transverse ridging/banding; excessive dryness, darkness in nails, curved nail ends	Iron, protein, vitamin B12
Head/Hair	Dull/lackluster; banding/sparse; alopecia; depigmentation of hair; scaly/flaky scalp	Protein and energy, biotin, copper, essential fatty acid
Eyes	Pallor conjunctiva	Vitamin B12, folic acid, iron
	Night vision impairment	Vitamin A
	Photophobia	Zinc
Oral cavity	Glossitis	Vitamins B2, B6, B12, niacin, iron, folic acid
	Gingivitis	Vitamin C
	Fissures, stomatitis	Vitamin B2, iron, protein
	Cheilosis	Niacin, vitamins B2, B6, protein
	Pale tongue	Iron, vitamin B12
	Atrophied papillae	Vitamin B2, niacin, iron
Nervous system	Mental confusion	Vitamins B1, B2, B12, water
	Depression, lethargy	Biotin, folic acid, vitamin C
	Weakness, leg paralysis	Vitamins B1, B6, B12, pantothenic acid
	Peripheral neuropathy	Vitamins B2, B6, B12
	Ataxia	Vitamin B12
	Hyporeflexia	Vitamin B1
	Muscle cramps	Vitamin B6, calcium, magnesium
	Fatigue	Energy, biotin, magnesium, iron

**Table 7:** Macronutrient requirements for adults.

Macronutrient	Energy Content/g	Recommended Amount/kg Body Weight/d
Proteins	4 kcal	1.0–1.5 g
Carbohydrates	4 kcal	max. 3–5 g
Fats	9 kcal	0.8–1.5 g

## Appendix A

**Table A1:** MNA full screening tool.

**Screening**		
A	Has food intake declined over the past 3 months due to loss of appetite, digestive problems, chewing or swallowing difficulties?	0 = severe decrease in food intake
		1 = moderate decrease in food intake
		2 = no decrease in food intake
B	Weight loss during the past 3 months	0 = weight loss greater than 3 kg
		1 = does not know
		2 = weight loss between 1 and 3 kg
		3 = no weight loss
C	Mobility	0 = bedridden or chair bound
		1 = able to get out of bed/chair but does not go out
		2 = goes out
D	Has suffered psychological stress or acute disease in the past 3 months?	0 = yes
		2 = no
E	Neuropsychological problems	0 = severe dementia or depression
		1 = mild dementia
		2 = no psychological problems
F1	Body mass index (BMI)	0 = BMI less than 19
		1 = BMI 19 to less than 21
		2 = BMI 21 to less than 23
		3 = BMI 23 or greater
**Screening Score** **(subtotal max. 14 points)**		
12–14 points	Normal nutritional status	
8–11 points	At risk of malnutrition	
0–7 points	Malnourished	
For a more in-depth assessment, continue with questions G-R		
**Assessment**		
G	Lives independently (not in nursing home or hospital)	0 = yes
		1 = no
H	Takes more than 3 prescription drugs per day	0 = yes
		1 = no
I	Pressure sores or skin ulcers	0 = yes
		1 = no
J	How many full meals does the patient eat daily?	0 = 1 meal
		1 = 2 meals
		2 = 3 meals
K	Selected consumption markers for protein intake	0.0 = if 0 or 1 yes
		0.5 = if 2 yes
		1.0 = if 3 yes
	● Meat, fish or poultry every day	Yes/No
	● ≥1 serving of dairy products (milk, cheese, yoghurt) per day	Yes/No
	● ≥2 servings of legumes or eggs per week	Yes/No
L	Consumes ≥2 servings of fruit or vegetables per day?	0 = yes
		1 = no
M	How much fluid (water, juice, coffee, tea, milk...) is consumed per day?	0.0 = less than 3 cups
		0.5 = 3 to 5 cups
		1.0 = more than 5 cups
N	Mode of feeding	0 = unable to eat without assistance
		1 = self-fed with some difficulty
		2 = self-fed without any problem
O	Self view of nutritional status	0 = views self as being malnourished
		1 = is uncertain of nutritional status
		2 = views self as having no nutritional problem
P	In comparison with other people of the same age, how does the patient consider his/her health status?	0.0 = not as good
		0.5 = does not know
		1.0 = as good
		2.0 = better
Q	Mid-arm circumference (MAC) in cm	0.0 = MAC less than 21
		0.5 = MAC 21 to 22
		1.0 = MAC greater than 22
R	Calf circumference (CC) in cm	0 = CC less than 31
		1 = CC 31 or greater
**Malnutrition Indicator Score**		
24–30 points	Normal nutritional status	
17–23.5 points	At risk of malnutrition	
<17 points	Malnourished	
